# Assessing the Impact of Obesity on Pregnancy and Neonatal Outcomes among Saudi Women

**DOI:** 10.3390/nursrep11020027

**Published:** 2021-04-24

**Authors:** Nadia Adwani, Howieda Fouly, Tagwa Omer

**Affiliations:** 1 Al-Thagher Hospital, Jeddah 22361, Saudi Arabia; nadwani77@hotmail.com; 2 Faculty of Nursing, Assiut University, Asyut 71115, Egypt; 3 College of Nursing, King Saud Bin Abdul-Aziz University for Health Sciences, Jeddah 21423, Saudi Arabia; omert@ksau-hs.edu.sa

**Keywords:** obesity, BMI, maternal outcomes, neonatal outcomes

## Abstract

*Background:* The rising prevalence of obesity has a significant impact on obstetrics practice regarding maternal and perinatal complications includes recurrent miscarriage, pregnancy-induced hypertension, preeclampsia, gestational diabetes, and prolonged labor. *Objective:* To assess the impact of obesity on pregnancy and neonatal outcomes among Saudi women. *Methods:* The study was conducted at King Abdul-Aziz Medical City, Jeddah. *Design*: A cross-sectional retrospective design. A total number of 186 participants were recruited from July to December 2018 according to eligibility criteria. *The* data were collected retrospectively by a review of the chart records of the labor and delivery department. *Results:* The mean (SD) age of participants was 31.94 (5.67) years old; two-thirds were in obesity class I. There was a significant association between obesity and pre-existing thyroid disease and induced hypertension class III. However, episiotomy showed that obesity class III was significantly different from obesity class II. *Conclusion:* This study concludes obesity affects the outcomes of pregnant Saudi associations between obesity and preeclampsia, perineal tears, and episiotomy variables, and other variables reflect no associations. *Recommendations:* Further studies are needed to generalize the results. This study endorses the pregnant women start the antenatal follow-up from 1st trimester so, the data will be available on the system for research.

## 1. Introduction

In recent years, obesity has begun to be considered a global health problem. It is the fifth leading cause of death worldwide. Obesity is a condition of abnormal and excessive fat accumulation in adipose tissue, leading to adverse health effects. The significant contributors to weight gain, which may eventually lead to obesity, are decreased physical activity, increased dietary fat intake, and genetic factors [1]. The rise in obesity is associated with advanced age, which becomes apparent when considering the decrease in older adults’ physical activity and metabolic processes. Marital status, high educational level, alcohol use, and high socioeconomic status are other factors associated with obesity [2].

Obesity is measured using various methods, including body mass index (BMI), waist circumference (WC), waist-hip ratio, skinfold, and percent body fat measurements. BMI is the most frequently used diagnostic tool in the current classification system of obesity [3]. It is calculated by dividing weight in kilograms by height in square meters. The World Health Organization (WHO) divided BMI values into six categories to define different body weights, from underweight to obesity. These categories are underweight (less than 18.5), average weight (18.5–24.9), overweight (25.0–29.9), obesity class I (30.0–34.9), obesity class II (35.0–39.9), and obesity class III (40.0 or greater) [4].

Overweight and obesity have become the most general nutritional problems globally, as they impose significant burdens on health care systems. Obesity affects 2.1 billion people (almost one-third) in the world. If the current trend continues, this figure may reach nearly half of the world’s adult population by 2030 [5]. Moreover, obesity is associated with multiple diseases and may result in the death of millions of people every year. Furthermore, the risk of non-communicable diseases (NCD), such as hypertension (HTN), type 2 diabetes mellitus (T2DM), dyslipidemia, and cardiovascular disease (CVD), increases dramatically with obesity. Besides, obstructive sleep apnea and osteoarthritis relate to obesity [6].

Females were to have a higher rate of obesity. Its prevalence doubled between 1980 and 2008, from 8% in 1980 to 14% in 2008. The highest incidence of overweight and obesity in 2013 was in North Africa and the Middle East, where more than 65% of reproductive-age females were overweight or obese [6]. According to a study of overweight and obesity in Saudi women of childbearing age, the following rates of obesity were found: 22.4% were obesity class I, 11.1% were obesity class II, and 6.6% were morbidly obese (obesity class III) [6]. Furthermore, Saudi women have exceptional obstacles that can predispose them to a sedentary lifestyle, such as the essential wearing of abaya or full-length overgarment in public, gender segregation, and activities that are primarily at home [7,8].

Maternal obesity is one of the central risk factors for adverse pregnancy outcomes, including gestational diabetes mellitus (GDM), operative delivery, and stillbirth [9]. The prevalence of hypothyroidism among Saudi pregnant women is 13%, most of them were in their third trimester [10].

Maternal obesity increases perinatal mortality, which increases the risk of perinatal death and preterm birth, macrosomia, congenital anomaly, childhood obesity, and stillbirth. Also, maternal obesity is related to a higher risk of cesarean deliveries and a higher incidence of anesthetic and postoperative complications. Another major complication is preeclampsia, a specific syndrome characterized by new onset of hypertension with proteinuria that occurs after 20 weeks gestation. The actual cause of preeclampsia is unknown, but it is estimated to affect 2 to 8% of all pregnancies [11,12].

The impact of obesity on pregnant women extends to the method of delivery. Previous studies reported two-thirds of 63.6% of obese women delivered by cesarean section, and there was no association between obesity or overweight and episiotomy. Obesity may be protective against the risk of third- and fourth-degree tears [13,14].

### Significance of the study

According to World Atlas data, Saudi Arabia is on the list of most obese countries in the world and ranked in the 15th most obese country, with an overall obesity rate of 33.7% [15]. The prevalence of obesity in pregnant women is increasing and is associated with pregnancy-related complications and their outcomes. Moreover, obesity affects the chance of conception and might decrease the response to fertility treatment. In Saudi Arabia, 68% of pregnant women were obese [12].

Even with the availability of updated data about the prevalence of obesity in Saudi Arabia, there is a lack of research conducted in Saudi Arabia about the outcome of obese pregnant women. Therefore, the study aimed to assess the impact of obesity on pregnancy and neonatal outcomes among Saudi women through the following objectives:

Describe the maternal and the neonatal outcomes for obese pregnant women, and

Compare the maternal and neonatal outcomes of obese pregnant women in different obesity classes. In addition to answering the following questions:

− What is the effect of obesity on the maternal outcomes among Saudi women?
− What is the effect of obesity on neonatal outcomes?

## 2. Methods

### 2.1. Study Design

This study a cross-sectional cohort study retrospective design, which involves collecting data at one point in time. The phenomena of the course were captured during one data collection period. Cross-sectional arrangements are especially appropriate for describing the status of phenomena or relationships among phenomena at a fixed point [9]. In retrospective design, the phenomenon observed in the present is linked to phenomena occurring in the past. This study described the outcomes of obese pregnancy during the antenatal, intrapartum period, and the neonatal outcome at the delivery time.

#### Setting

This study was conducted in the labor room at King Khaled Hospital in Jeddah. The hospital affiliated to the Ministry of National Guard Health Affairs in the Western Region is a 600 bedded, Joint Commission International (JCI) accredited Tertiary Level Hospital. Currently, ten admission beds within the labor and delivery (6 beds for active labor, three beds for induction of labor, and one bed for operation room). The total number of deliveries per year was 3161.

### 2.2. Participants

All obese pregnant women of primigravida and multigravida, aged from 18 to 44 years old, and the BMI for those pregnant women was 30 or more. All pregnant attended the antenatal clinic during the first trimester and delivered through expected vaginal delivery at King Khaled Hospital, “during the selected six months” of the data collection period. The study excluded all multiple pregnancies and pre-pregnant diabetes mellitus, chronic hypertension before pregnancy, had a history of cardiac diseases, or the current pregnancy with abnormal fetus lie like a breech or transverse lie. All elective cesarean sections are excluded if it is related to the different cause than obesity.

### 2.3. Sample Size

**Figure 1:**
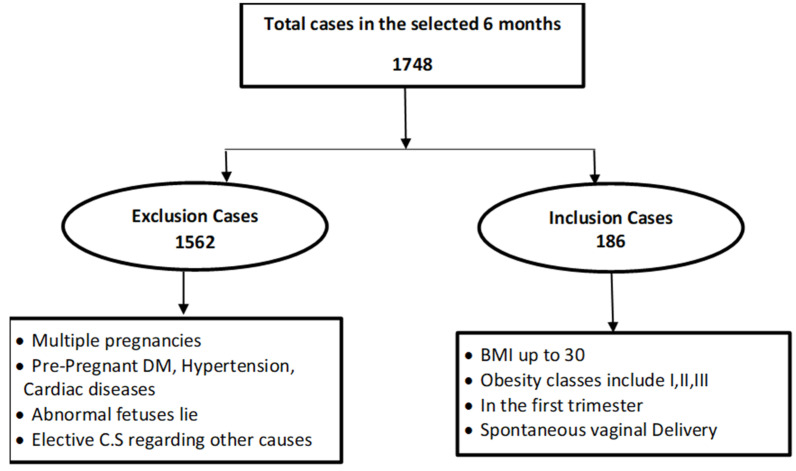
Flowchart of the recruited sample.

The total number of deliveries in the selected six months, during the period from 1 July 2018 to 31 December 2018, was 1748 delivery. After excluding the patients of pregnant women who did not meet the criteria, the total number of patients included in this study were 186 patients, which is the total sample (Figure 1).

### 2.4. Data Collection

Data was collected through a nonprobability sampling retrospectively by reviewing the chart records of the labor and delivery department. The study included all obese women who delivered in the selected six months. Then compared the outcomes of pregnancy through the different obesity’ classes.

The data was collected from the chart using the BEST Care 2.0 system, Initially, BESTCare has successfully went live in the central region, Riyadh, Saudi Arabia, represented by King Abdullah Specialist Children’s Hospital and King Abdulaziz Medical City early this year in January 2016, followed by the Go-Live in the western region in King Abdulaziz Medical City—Jeddah in May 2016. In August 2016, the BESTCare Go-Live took place in Prince Mohammed Bin Abdul Aziz Hospital - and founded in all the hospital computers which affiliated to the Saudi Korean health informatics company of information technology (SKHIC) Jeddah, Saudi Arabia.

### 2.5. Data Collection Instrument

The study checklist assesses the effect of obesity on pregnancy and fetal outcome was developed after extensive searching of the literature [6,10,16,17]. The most common complication for obese pregnant women during the antenatal period, intrapartum period, and the neonatal outcome at the time of delivery was listed. Then the sociodemographic and health-related characteristics were added and categorized with the maternal and neonatal outcomes. After the primary checklist was portrayed, eight experts’ King Saud Bin Abdulaziz University for Health Sciences, college of nursing faculty reviewed the checklist. They gave their opinion about it and what needs to be added or what to delete. Then the final form of the checklist formulated and de-signed into three main categories:

1. The first category is sociodemographic and health-related characteristics which include eight subcategories: BMI, maternal age, primigravida, multigravida, mode of the previous delivery, smoking in the current pregnancy, pre-existing thyroid disease, and recurrent miscarriages.
2. The second category is the maternal outcomes which divided into two sections:
  - The antenatal complication includes four subcategories: Pregnancy-induced hypertension (preeclampsia or eclampsia), gestational diabetes, venous thromboembolism, and urinary tract infection.
  - The intra-natal outcomes include twelve subcategories: Gestational age at delivery, preterm labor, induction of labor, augmentation of labor, mode of current delivery, perineal tears (first-degree second degree and third-degree), perineal episiotomy, placental complete or incomplete, duration of 3ed stage of labor, emergency cesarean delivery, postpartum hemorrhage, and prolonged labor.
3. The third category is the neonatal outcomes, which includes ten subcategories: Intrauterine growth restriction IUGR, intra-uterine fetal death IUFD, congenital anomalies, preterm baby, shoulder dystocia, stillbirth, APGAR score, neonatal mortality, birth weight (appropriate for gestational age [AGA]-small for gestational age [SGA]-large for gestational age [LGA]) and admission to NICU.

### 2.6. Validity and Reliability

The checklist was reviewed by eight of the nursing college’s expertise and faculty members, King Saud bin Abdulaziz University for health sciences. It is validated through content and face validity, which refers to whether the instrument looks as though it measures the appropriate construct, especially to people who will be completing the tool [18].

### 2.7. Data Management

The data analyzed using the Statistical Package of Social Sciences (SPSS, IBM, Armonk, NY, USA) version 23. Proper statistical tests were used to describe the finding of the study and to achieve the objectives of the study, with appropriate statistical measures and tests. The ordinal and nominal variables were presented in the form of frequencies and percentages. Chi-square, which is a statistical test, was used to examine the association between two quantitative variables. *p*-value of less than 0.05 was considered statistically significant, and it can be concluded that there is a relationship between the two variables. Fisher’s exact test was performed to test the significance of the difference in proportions; it is used for small sample size or when the cells in the contingency table have no observations. Post hoc test was also used for comparing all possible pairs of groups following a significant test of overall group differences (e.g., chi-square) [18] (Table S1).

### 2.8. Ethical Considerations

Ethical codes conducted are to be strictly adhered to at all stages of the study. Before implementing the project, the proposal was submitted to the College of Nursing Research unit for a review then submitted to King Abdullah International Medical Research Center (KAIMRC). The approval from KAIMARC was received for the study number SP19/114/J on (G) 21 April 2019, (H) 16th Shaban 1440. Then, the Institutional Review Board (IRB)’s approval for the research study received on 5 May 2019 and valid for one year. Memo ref.NO. IRB C/0613/19, E-CTS ref.NO. JED-19-427780-71475. IRB NCBE registration No: H-01-R-005. Also, the hospital agreement was obtained before data collection started.

Confidentiality, privacy, and anonymity were maintained. This study dealt with chart records, so if there is any hard copy, personal information or identities will be kept safe in a locker. This data will be held for the study for a minimum of five years and destroyed after that.

## 3. Results

Table 1 presents respondents’ essential characteristics; the majority are 28.5% of women aged between 25 to 29 years, and only 12.4% for the age group between 40 to 45 years. Regarding the obstetric characteristics, results revealed that primigravida was only 16.7%, while the majority were multigravida’ 83.3%. The mode of previous delivery 67.7% were delivered through SVD and 14.0% paid by CS. Two-thirds of women were in class I obese (BMI 30–34), while 7.5% of them in obesity class III (BMI more than 40). Regarding the risk factors, only 2.2% were smokers, 8.6% have pre-existing thyroid disease, and 5.4% had recurrent miscarriages.

Table 2 shows the frequency and result of fisher’s exact test that examined the relationship between obesity classes and co-factors. The pre-existing thyroid disease shows that 16 (12.5%) cases were found in obesity class I. There was a significant association between obesity and pre-existing thyroid disease at a 95% level of confidence, and *p =* 0.015. Only one case (7.1%) and 14 (92.9%) were found in class III obesity for preeclampsia. The association between obesity and preeclampsia at a 90% confidence level reflected no statistically significant at *p* = 0.075. Similarly, there is no statistically significant difference between (GDM) and UTI with obesity. For perineal tears, intact perineal at delivery was 55 (43.0%) cases, while 45 (35.2%) 24 (18.8%) had first and second-degree tears, respectively. For obesity class II, 24 women (54.5%) deliver with an intact perineum, 13 (29.5%) and 4 (9.1%) cases had 1st and second-degree tears, respectively. In obesity class III, the highest percentage, 6 (42.0%) deliver with intact perineal, while tears were 4 (28.6%), and (7.1%) cases had first and second-degree tears, respectively. There is a significant association between obesity and perineal tears at a 95% confidence level and *p =* 0.020. The association between episiotomy and obesity reflected that class was 3 (6.8%) cases out of 44 (23.7%) and class III, 3 (21.4%) out of 14 (7.5%) had an episiotomy. There is a significant association between obesity and episiotomy at a 95% level of confidence. The P-value of the test is 0.037.

Table 3 shows the frequency and result of fisher’s exact test that examined the relationship between obesity classes and neonatal outcomes. The gestational age categories Preterm10 (7.8)%.

Full term117 (91.4) and Post date1 (0.8) relationship with obesity class I at *p* < 0.325. Similarly, class II& III reflected no statistically significant relationship. All other co-factors regarding neonatal outcomes of IUFD, Preterm babies, Apgar score, birth weight, admission to NICU, and neonatal mortality in relation to obesity classes reflected no statistical significant relationship at (*p* < 0.751, *p* < 1.000, *p* < 1.000, and *p* < 0.312) respectively.

Table 4 Shows the result of post-hoc tests that were conducted to test pairwise comparisons. The finding indicates that Pre-existing thyroid disease compared to obesity class I was significantly different from obesity class II (*p* = 0.033). Obesity class I and obesity class III, and both obesity class II and class III were not significantly different.

The comparison of induced hypertension shows that obesity class III was significantly different from obesity class I (*p* = 0.001). Furthermore, obesity class III was significantly different from obesity class II (*p* = 0.001). On the other hand, obesity class I and class II were not significant in regard to induced hypertension. The finding of perineal tears shows that obesity class I was significantly different from obesity class III (*p* = 0.020). Either obesity class I and obesity class II or obesity class II and class III were not significantly different. The result of episiotomy shows that obesity class III was significantly different from obesity class II (*p* = 0.044). Also, obesity class III was significantly different from obesity class I (*p* = 0.008). Besides, there is no statistically significant difference between obesity class II and class I.

## 4. Discussion

The rising prevalence of obesity has a significant impact on obstetrics practice. Maternal complication association due to obesity includes recurrent miscarriage, pregnancy-induced hypertension, preeclampsia, gestational diabetes, prolonged labor, and increased risk of interventions like induction of labor, operative delivery, shoulder dystocia, and postpartum hemorrhage [13]. On the other hand, perinatal complications include congenital disabilities like congenital anomalies, macrosomia, stillbirth, preterm birth, and the need for admission to the neonatal intensive care unit [13].

Our study aimed to assess the impact of obesity on pregnancy and neonatal outcomes among Saudi women. This study’s first objective was to describe the pregnancy and the neonatal outcomes for obese pregnant women. The present study revealed a significant association between obesity and pre-existing thyroid disease, pregnancy-induced hypertension (preeclampsia), perineal tears, and episiotomy.

Regarding the association between obesity and pre-existing thyroid disease, our result shows a statistically significant association between obesity and pre-existing thyroid disease. Similarly, in a prospective cohort study [19] conducted at Rotterdam, the Netherlands. The study aimed to examine maternal thyroid function associations in early pregnancy with maternal BMI and weight gain during pregnancy. This study identified a higher maternal thyroid stimulating hormone (TSH) level in early pregnancy associated with a higher pre-pregnancy BMI and an increased risk of excessive gestational weight gain. In contrast, a higher maternal FT4 level was associated with a lower pre-pregnancy BMI and a lower risk of excessive gestational weight gain. Associations of maternal thyroid function with gestational weight gain were strongest for weight gain in early pregnancy. Also, [20] in a prospective follow-up study of thyroid parameters and gestational weight gain, indicated that higher median TSH and lower median FT4 levels in all trimesters were correlated with a higher amount of total weight gain during pregnancy.

Conversely, a study conducted at Baylor aimed to measure T3, FT4, and TSH in maternal and matched cord blood serum from average weight, overweight and obese gravidae to determine alterations in maternal and neonatal TH levels by maternal obesity. The result showed no significant difference in gestational age and weight gain [21].

Regarding the association between obesity and preeclampsia, our result shows a statistically significant association between obesity and preeclampsia, similarly, in prospective cohort research conducted in Jeddah Maternity and Children Hospital (MCH). The study revealed a positive association between obesity and increased risk of pregnancy-induced hypertension and preeclampsia compared with the normal-weight women. A previous study by [21] conducted in Tabuk City found a higher risk of preeclampsia in obese non-GDM women.

Likewise, [22] demonstrated in the retrospective cohort study that women with pre-pregnancy obesity are more likely to develop preeclampsia, which reported a statistically significant association between obesity and preeclampsia.

According to [23], in a review that aimed to summarize the findings of published systematic reviews regarding the possible risks for pregnant women with obesity and their infants. The review demonstrates an association between obesity and gestational hypertension and preeclampsia, identified as a risk factor in 54 studies.

A cohort study was done by [24], where data was collected from three large urban academic centers; the result revealed a positive association between obesity and preeclampsia. Moreover, a study by [25] reported in the systematic literature review of two decades (1992–2011) that obesity is associated with preeclampsia or hypertension during pregnancy.

On the other hand, the findings reported by [26] in a prospective cohort study, the result shows that higher gestational weight gain was associated with a higher risk of pregnancy-induced hypertension but not with preeclampsia. Additionally, higher pre-pregnancy BMI is associated with high blood pressure, both systolic and diastolic, in all trimesters.

Regarding perineal tears, our result shows a statistically significant association between obesity and perineal tears. the association between these two variables is a significant negative association, which means when the obesity class decreases, the risk for perineal tears increased. Conversely, when the obesity class increased, the risk for perineal tears decreased.

Similarly, [14] in the case-control study, revealed no significant association between obesity and perineal tears. Likewise, a previous cohort study by Blomberg, 2014 [27] which demonstrated that the risk of partial anal sphincter injury or total sphincter injury and fourth-degree perineal tears decreased with maternal obesity. The general risk for any anal sphincter injury among obese women class III was reduced by 25% compared to normal women’s weight.

Previous studies found that obese women have a lower risk for perineal tears, known as a protective effect of obesity. The negative association between obesity and perineal tears has shown that obesity in pregnancy is not commonly associated with adverse events [28].

A retrospective cohort study [29] conducted in Riyadh, Saudi Arabia, indicated an association between obesity and perineal tears. Furthermore, the prospective cohort study [30] which was carried out in Egypt, reported that obese women had a higher perineal tear rate, mostly second-degree tearing, than those with normal BMI. Besides, there was no significant difference in the incidence of third-degree perineal tear in obese women.

In the same line, [31] found that the rate of third- and fourth-degree perineal tears decreased with increasing BMI, whereas the opposite was true for first- and second-degree perineal tears, which increased with increasing BMI.

In conclusion, the incongruence between our study and those studies may be due to the large study population, and the data included an ethnically heterogeneous population.

Regarding episiotomy, our result shows a statistically significant association between obesity and episiotomy. Dissimilarly, the case-control study was conducted on pregnant women and reported no association between obesity and episiotomy [15].

In the same line, [32] reported in a prospective cohort study conducted at Bingham. that there was no increased risk of episiotomy/perineal tear among obese pregnant women.

Similarly, a systematic review and meta-analysis were conducted to investigate maternal obesity in Africa [33] and reflected no significant relationship between maternal obesity and episiotomy or perineal tear. The contrast between our study and those studies may be due to the ethnically heterogeneous population.

This study’s second objective was to compare the pregnancy and the neonatal outcomes of obese pregnant women in different obesity classes. Regarding preeclampsia, our study’s findings were in accordance with other findings reported by [23] which stated that preterm preeclampsia is high in women with class III obesity compared to normal-weight women. This agrees with only one case in our study, which was categorized under obesity class III.

Several previous studies [4,23,24,25,33,34] reflected their result based on a comparison of obesity in general with average weight not like our study the comparison in obesity classes. Obese women have an increased risk of gestational diabetes and giving birth to macrocosmic children regardless of their glycemic status. To limit the pregnancy complications of obesity, it is common obstetric practice to restrict weight gain in obese women with diet/physical exercise. These factors may have influenced the fetal growth, and thus, the consequent risk of perineal tears/episiotomy/cesarean sections. In this study, neither the prevalence rates of gestational diabetes nor the amount of gestational weight gain was addressed [35,36].

## 5. Limitation of the Study

Missing data inpatient medical record led to exclude the patients from the sample. The exclusion criteria decrease the sample size, which affects reversibility. Also, the lack of a normal-weight control group would have provided the baseline rates of obstetrical complications in Saudi women, which is not included in our study.

### Generalizability

To find the relationship between obesity and labor outcome, the study needs to extend and be conducted on a larger sample to generalized in the future.

## 6. Conclusions

The study sheds light on a significant risk factor among Saudi pregnant women as obesity is a severe public health problem and harms maternal pregnancy outcomes. This study reflected several associations between obesity and specific co-factors as preeclampsia, perineal tears, and episiotomy variables.

## 7. Implication for Future Practice

The findings of the study draw a primary direction of essential data that should be included in the care system to prevent the future complication of obesity among pregnant women.

## 8. Recommendations

Further studies are needed to conduct a larger sample size and include the elective cesarean section and its relation to obesity. This study encourages the broadness of data to have all Jeddah districts, not only one point, and increases the data collection period to generalize the results. The study also recommends the pregnant women start the antenatal follow-up from 1st trimester so that the data will be available on the research system.

## Figures and Tables

**Table 1:** The Sociodemographic characteristics of study’s participants.

Variable	Categories	N = 186	%
Age	Less 24	15	8.1
	25–29	53	28.5
	30–34	50	26.9
	35–39	45	24.2
	40–45	23	12.4
Primigravida	Yes	31	16.7
Multigravida	Yes	155	83.3
Previous delivery	SVD (Spontaneous vaginal delivery)	126	67.7
	CS (Caesarean section)	26	14.0
Obesity	(Primigravida)	31	16.7
	Class I (BMI 30–34)	128	68.8
	Class II (BMI 35–39)	44	23.7
	Class III (BMI more than 40)	14	7.5
Current Smoking	Yes	4	2.2
Pre-existing thyroid diseaseRecurrent miscarriages	No	182	97.8
	Yes	16	8.6
	No	170	90.4
	Yes	10	5.4
	No	176	93.6

**Table 2:** Association between Obesity and obstetrics co-factors.

KERRYPNX	Obesity			
	Class I (BMI 3–34 kg/m^2^) *n* = 128 (%)	Class II (BMI 35–39.9kg/m^2^) *n* = 44 (%)	Class III (BMI > 40.0 kg/m^2^) *n* = 14 (%)	*p* Value *
Pre-existing thyroid disease				0.015 *
Yes	16 (12.5)	0	0	
No	112 (87.5)	44 (100)	14 (100)	
Pregnancy induced hypertension				0.075
Yes	0	0	1 (7.1)	
No	128 (100)	44 (100)	13 (92.9)	
Gestational diabetes				0.624
Yes	29 (22.7)	12 (27.3)	12 (27.3)	
No	99 (77.3)	32 (72.7)	32 (72.7)	
Urinary tract infection				0.528
Yes	1 (0.8)	1 (2.3)	0	
No	127 (99.2)	43 (97.7)	14 (100)	
Perineal tear				0.020 *
First degree	45 (35.2)	13 (29.5)	1 (7.1)	
Second degree	24 (18.8)	4 (9.1)	4 (28.6)	
Third degree	1(0.8)	0	0	
Intact	55 (43.0)	24(54.5)	6(42.9)	
Episiotomy				0.037 *
Yes	5 (3.9)	3 (6.8)	3(21.4)	
No	123 (96.1)	41 (93.2)	11 (78.6)	
Induction of Labor	33 (25.8)	11 (25.0)	5 (35.7)	0.730
Augmentation of labor	26 (20.3)	9 (20.5)	6 (42.9)	0.163
Method of Delivery				
spontaneous vaginal delivery (SVD)	97 (75.8)	31 (70.5)	9 (64.3)	0.505
Instrumental	8 (6.3)	2 (4.5)	0	
Caesarean section (CS)	23 (18.0)	11 (25.0)	5 (35.7)	0.112
Labor complications				
postpartum haemorrhage				
Yes	3 (2.3)	1 (2.3)	1 (7.1)	0.399

* Statistically significant *p <* 0.05.

**Table 3:** Association between Obesity and Neonatal outcomes.

	Obesity			
	Class I (BMI 30–34 kg/m^2^) *n* = 128(%)	Class II (BMI 35.0–39.9kg/m^2^) 44 (%)	Class III (BMI—40.0 kg/m^2^) *n* = 14 (%)	*p* Value *
Gestational age				0.325
Preterm	10 (7.8)	3 (6.8)	0	
Full term	117 (91.4)	41 (93.2)	13 (92.9)	
Post date	1 (0.8)	0	1 (7.1)	
IUFD	0	1 (2.3)	0	0.312
Preterm baby	10 (7.8)	3 (6.8)	0	0.894
APGAR score				0.751
0–2 need resuscitation	1 (0.8)	1 (2.3)	0	
3–6 stimulation	3 (2.3)	1 (2.3)	0	
7–10 no action	124 (96.9)	42 (95.5)	14 (100)	
Birth weight				1.000
AGA (Appropriate for gestational age)	108 (84.4)	38 (86.4)	13 (92.9)	
SGA (Small for gestational age)	12 (9.4)	4 (9.1)	1 (7.1)	
LGA (Large for gestational age)	8 (6.3)	2 (4.5)	0	
Admission to NICU	4 (3.1)	1 (2.3)	0	1.000
Neonatal mortality	0	1 (2.3)	0	0.312

* Statistically significant *p* < 0.05 ** Statistically significant *p* < 0.01.

**Table 4:** Comparison of obesity classes within Pre-existing thyroid disease.

Obesity	Test Statistic	Pre-Existing Thyroid Disease	*p* Value
	Class I-class II	−11.625	0.033 *
	Class I-class III	−11.625	0.343
	Class II-class III	0.000	1.000
**Obesity**		**Induced Hypertension** **Test Statistic**	***p* Value**
	Class III-class I	6.643	0.001 **
	Class III-class II	6.643	0.001 **
	Class I-class II	0.000	1.000
**Obesity**		**Perineal tears** **Test Statistic**	***p* Value**
	Class I-class II	−14.077	0.108
	Class I-class III	−32.876	0.020 *
	Class II-class III	−18.799	0.222
**Obesity**		**Episiotomy** **Test Statistic**	***p* Value**
	Class III-class II	13.588	0.044
	Class III-class I	16.296	0.008 *
	Class II-class I	2.708	0.481

* Statistically significant *p* < 0.05 ** Statistically significant *p* < 0.01.

## Data Availability

Data used and analyzed in this study will be promptly available for the publisher upon request.

## Supplementary Materials

The following are available online at https://www.mdpi.com/article/10.3390/nursrep11020027/s1, Table S1: STROBE Statement—Checklist of items that should be included in reports of ***cross-sectional studies***.
